# A Case Series Describing the Recurrence of COVID-19 in Patients Who Recovered from Initial Illness in Bangladesh

**DOI:** 10.3390/tropicalmed6020041

**Published:** 2021-03-31

**Authors:** Pritimoy Das, Syed M. Satter, Allen G. Ross, Zarin Abdullah, Arifa Nazneen, Rebeca Sultana, Nadia Ali Rimi, Kamal Chowdhury, Rashedul Alam, Shahana Parveen, Md Mahfuzur Rahman, Mohammad Enayet Hossain, Mohammed Ziaur Rahman, Razib Mazumder, Ahmed Abdullah, Mahmudur Rahman, Sayera Banu, Tahmeed Ahmed, John D. Clemens, Mustafizur Rahman

**Affiliations:** International Centre for Diarrhoeal Disease Research, Bangladesh (icddr,b), Dhaka 1212, Bangladesh; dr.satter@icddrb.org (S.M.S.); allen.ross@icddrb.org (A.G.R.); zarin.abdullah@icddrb.org (Z.A.); arifa.nazneen@icddrb.org (A.N.); rebeca@icddrb.org (R.S.); nadiarimi@icddrb.org (N.A.R.); kiachowdhury@icddrb.org (K.C.); rashedul.alam@icddrb.org (R.A.); sparveen@icddrb.org (S.P.); mf.rahman@icddrb.org (M.M.R.); enayet.hossain@icddrb.org (M.E.H.); mzrahman@icddrb.org (M.Z.R.); rmazumder@icddrb.org (R.M.); ahmed.abdullah@icddrb.org (A.A.); rahman.mahmudur@icddrb.org (M.R.); sbanu@icddrb.org (S.B.); tahmeed@icddrb.org (T.A.); jclemens@icddrb.org (J.D.C.); mustafizur@icddrb.org (M.R.)

**Keywords:** COVID-19, recurrence, reinfection, Bangladesh

## Abstract

To date, severe acute respiratory syndrome coronavirus-2 (SARS-CoV-2) has infected over 80 million people globally. We report a case series of five clinically and laboratory confirmed COVID-19 patients from Bangladesh who suffered a second episode of COVID-19 illness after 70 symptom-free days. The International Centre for Diarrhoeal Disease Research, Bangladesh (icddr,b), is a leading public health research institution in South Asia. icddr, b staff were actively tested, treated and followed-up for COVID-19 by an experienced team of clinicians, epidemiologists, and virologists. From 21 March to 30 September 2020, 1370 icddr,b employees working at either the Dhaka (urban) or Matlab (rural) clinical sites were tested for COVID-19. In total, 522 (38%) were positive; 38% from urban Dhaka (483/1261) and 36% from the rural clinical site Matlab (39/109). Five patients (60% male with a mean age of 41 years) had real-time reverse transcription-polymerase chain reaction (rRT-PCR) diagnosed recurrence (reinfection) of SARS-CoV-2. All had mild symptoms except for one who was hospitalized. Though all cases reported fair risk perceptions towards COVID-19, all had potential exposure sources for reinfection. After a second course of treatment and home isolation, all patients fully recovered. Our findings suggest the need for COVID-19 vaccination and continuing other preventive measures to further mitigate the pandemic. An optimal post-recovery follow-up strategy to allow the safe return of COVID-19 patients to the workforce may be considered.

## 1. Introduction

SARS-CoV-2, a novel enveloped RNA beta coronavirus, has manifested a variety of clinical characteristics from asymptomatic infection to severe pneumonia, vasculitis and death [1,2,3]. The World Health Organization (WHO) declared this disease a pandemic on 11 March 2020. As of 29 December 2020, SARS-CoV-2 infected more than 81 million people worldwide, with 1,784,533 deaths [4]. Bangladesh officially declared its first COVID-19 case on 8 March 2020 [5]. By 30 December 2020, 512,496 cases and 7531 deaths were reported in the country [6]. 

Direct, indirect or close contact with infected people and exposure to their saliva and respiratory droplets, which are released when an infected person coughs, sneezes, or speaks, may cause viral transmission [7]. The median incubation period of this virus is about 5 days and the infectious period duration is approximately 10 days [8,9]. A meta-analysis found that 59% of all transmission came from asymptomatic carriers [8].

WHO defines a laboratory confirmed case of COVID-19 if the patient’s sample (at least a single nasopharyngeal (NP) and/or oropharyngeal swab or wash and/or lower respiratory sputum and/or endotracheal aspirate or bronchoalveolar lavage) tests positive by real-time reverse transcription-polymerase chain reaction (rRT-PCR) [10]. rRT-PCR assays only inform clinicians whether SARS-CoV-2 is present or not. However, these assays also provide quantitative data on cycle threshold (Ct) values, which are inversely related to the viral load and are not reported clinically. An advanced assay (e.g., whole genome sequencing) is required to interpret a recurrence—whether it is a relapse or a true reinfection. The effect of SARS-CoV-2 viral load on clinical outcomes and recurrence has not been extensively studied. 

Generally, “relapse” may be defined as a “recurrence” with the same species and strain of a micro-organism that was present before, whereas “reinfection” is a secondary infection with a different species or strain. Reinfections with respiratory viruses may occur as a result of a weakened or waning immune response (e.g., respiratory syncytial virus), reinfection with a different genotype/species (e.g., rhinoviruses), or the viruses’ high variability (e.g., influenza virus). The immunity against SARS-CoV-2 is not well established and it is uncertain how long the antibody will prevent re-infection [11]. Moreover, vaccine-induced protective immunity may differ from natural immunity due to the immune-evasion strategies of wild-type viruses [12]. However, there is some evidence of persistence of neutralizing antibodies in COVID-19 patients with natural infection for the first few months (~8 months) [13,14]. The immune response following a natural infection is thought to be incomplete, and reinfections are likely [15]. One should not confuse relapse with “long COVID” which refers to symptoms of COVID-19 that continue after the typical convalescence period. According to some reports, about 10% of people who tested positive for SARS-CoV-2 had one or more symptoms for more than 12 weeks [16].

Reinfection with SARS-CoV-2 is rare but possible, according to recent studies. Positive RT-PCR results in patients who recovered from initial illness have been reported in a number of countries [17]. In August 2020, a reinfection case was confirmed in Hong Kong in a patient with clearly different genome sequences [18]. Three confirmed reinfection cases were also reported from Nevada, USA [19], Belgium [20] and Ecuador [21].

There are some anecdotal reports of reinfection/relapse from Bangladesh. Among the positive COVID-19 staff cases from the International Centre for Diarrhoeal Disease Research, Bangladesh (icddr,b), the investigators noticed that some patients who clinically recovered from their illness (RT-PCR negative) were found to be RT-PCR positive for SARS-CoV-2 a second time. The aim of our study was to investigate suspected cases of reinfection or relapse excluding long COVID. We explored the clinico-epidemiological data of recurrent COVID-19 cases in Bangladesh and correlated these findings with their RT-PCR test results and whole genome sequencing.

## 2. Materials and Methods

### 2.1. Study Design and Setting

We conducted a case series of icddr, b staff between March and September 2020 where the community transmission of SARS-COV-2 was established.

### 2.2. Study Population and Procedure

The International Centre for Diarrhoeal Disease Research, Bangladesh (icddr,b) is a leading public health research institution in South Asia with approximately 4000 staff. Since 21 March 2020, all staff with clinical features (fever, cough, cold or respiratory distress) of COVID-19 were instructed to contact the icddr,b Staff Clinic. The staff clinic doctors advised any suspected staff member to undertake a nasopharyngeal swab for a COVID-19 test using RT-PCR. Some high-risk staff, such as staff nurses collecting NP swabs and laboratory personnel working with SARS-CoV-2, were also advised to undertake routine COVID-19 tests.

When a patient was RT-PCR positive, staff clinic doctors advised patients for home isolation if the condition was not severe enough for hospitalization. The staff clinic also prescribed and distributed medicines for each COVID-19 positive patient. Along with severe cases, less severe cases were also admitted to the isolation center of icddr,b if the staff member had difficulty with home isolation. All positive cases were advised to have a repeat test on day 14 and every week thereafter until tested negative.

The WHO criteria [22] for withdrawing from isolation for symptomatic patients as: 10 days after symptom onset, plus at least three additional days without symptoms (including fever and respiratory symptoms); and for asymptomatic cases: 10 days after a positive test for SARS-CoV-2. Based on the WHO case criteria and recent publications [23], we set the following criteria for selecting patients to investigate for reinfection: (1) an initial SARS-CoV-2 PCR-confirmed diagnosis; (2) followed by clinical recovery and with at least one negative SARS-CoV-2 PCR result; (3) followed by a confirmed SARS-CoV-2 PCR positive result from a single nasopharyngeal swab test with clinical symptoms at least 28 days after the previous SARS-COV-2 negative PCR result. The WHO clinical progression scale [24] specific for COVID-19 was used for clinical classification and assessing disease severity.

### 2.3. Laboratory Investigations

All participants submitted a single nasopharyngeal swab that was collected by trained nurses in viral transportation media. The specimens were transported to the laboratory in coolers within an hour of collection. RNA was extracted from the nasopharyngeal samples using QiaAmp Viral RNA Mini kit (Qiagen, Hilden, Germany). A final volume of 60 microliters of RNA was eluted from a 140-microliter sample. RNA was tested for SARS-CoV-2 by real-time reverse transcription polymerase chain reaction (rRT-PCR) targeting ORF1ab- and N-gene specific primers and probes following the protocol of Chinese Center for Disease Control and Prevention (briefly as China CDC). A positive case was determined if the CT values of two targets (ORF1ab and N) were <37 in the same specimen. If CT values of any sample were 37−40 or a single target was positive, it was resampled and retested. If the CT values were still 37−40 and the amplification curves had obvious peaks, the sample was considered positive. We did repeat tests for all second-time positives to confirm that they were not false-positive cases. If a single target was positive in the repeat test, it was determined to be positive. The complete sequencing of positive isolates from the first and second episodes of each case was conducted using the Illumina NextSeq 500 platform. The RNA libraries were prepared using the Illumina TruSeq Stranded Total RNA Gold Library kit (Illumina, San Diego, CA, USA) following the manufacturer’s instructions. The normalized pooled library was sequenced employing the Illumina NextSeq v2.5 sequencing kit (Illumina, San Diego, CA, USA). Additionally, all samples were tested for common respiratory viruses including: influenza A and B viruses, respiratory syncytial virus (RSV), parainfluenza (1, 2, 3), human metapneumovirus (hMPV), and adenovirus using real-time PCR following standard procedure [25,26].

## 3. Results

From 21 March to 30 September 2020, among the 3056 icddr,b employees working at Dhaka and Matlab centers, 1370 were tested for COVID-19 and 522 (38%) tested positive. Within this period, eight patients (1.5%) had recurrent episodes of COVID-19 infection. Three were excluded due to a short gap (less than 28 days) between previous PCR negativity and subsequent PCR positivity and the absence of any clinical sign/symptoms during their first episode of COVID-19 positivity. None of them had a history of compromised immunity. Of the five remaining patients who fulfilled our criteria for selecting patients to investigate for reinfection, 60% were male. All five cases were 35 to 49 years old with a mean age of 41 years (Table 1). All five cases perceived that the second episode, which occurred after a long recovery time, was a COVID-19 reinfection.

**Figure 1:**
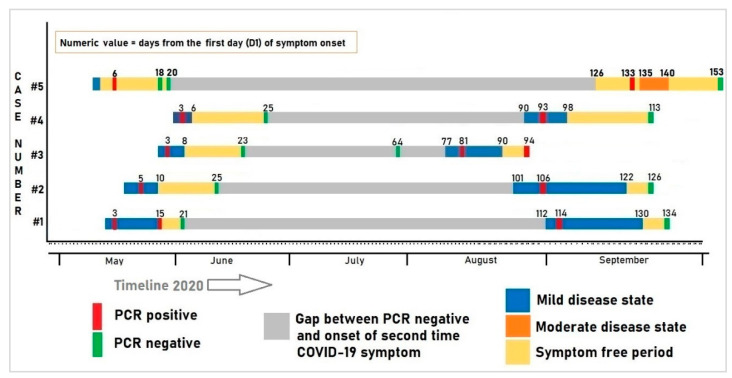
Clinical course and the corresponding PCR test results of the five patients with recurrent episodes of COVID-19 infection.

The clinical course of the patients over time (onset of symptoms, severity, duration, recovery time) and PCR results are illustrated in Figure 1. Figure 1 clearly demarcates two episodes of COVID-19 illness.

### 3.1. Case Series

#### 3.1.1. Case 1

The first case presented with fever and a cough starting on 13 May (D1; D = day). The nasopharyngeal swab was drawn on 16 May, and the RT-PCR test was COVID-19 positive. The patient had a history of hypertension for three years. The patient reported no contact with suspected cases. He was advised to home isolate and was treated with azithromycin, ivermectin, doxycycline and zinc tablets (Table 1). He became asymptomatic on D15. On 3 June, his RT-PCR was negative. After a 98-day asymptomatic period, he developed a fever, dry cough and cold on 2 September, and tested positive a second time on 4 September. Case 1 reported attending a meeting at his office and also shopping at a nearby wet market for fish and other groceries in the 14 days before the onset of the second time illness. Although the case reported wearing a mask and using hand sanitizer during these outside visits, he states that these visits may have been the source of COVID-19 exposure. His symptoms persisted for 18 days before full clinical recovery and his RT-PCR was negative 22 days after the second onset of symptoms (24 September). He was well aware of COVID-19 as he read extensively about the infection from different online materials. In terms of vaccination, he was hopeful, assuming that it would continue to reduce the risk of COVID-19 and its severity. His test results are summarized in Table 2.

#### 3.1.2. Case 2

The second case was a research assistant. She reported malaise on 19 May (D1). Two days later, her RT-PCR test was found to be positive. She had no other comorbidity. She went only to her workplace in the past 14 days before the onset of symptoms. The patient reported no contact with suspected cases. She was advised to home isolate. Her ECG, blood pressure and oxygen saturation were within normal limits. She became asymptomatic after D10 (27 May). She was tested again on D25 (12 June) and her RT-PCR test was negative. Case 2 reported that she went to the office regularly after being PCR negative. She used public transportation to and from work as well as on field trips. After a 92-day asymptomatic period, she developed a sore throat, fever, dry cough and a headache on 27 August. She was retested on 1 September and was found to be RT-PCR positive for the second time. No family members or any close contacts were positive for coronavirus within this time frame. She reported that she might have unknowingly been exposed to someone who is COVID-19 positive while traveling or engaging with patients and their caregivers at her workstation. For the second episode of illness, she was again treated at home with oral medication (Table 1). Her symptoms persisted for 21 days and her RT-PCR test was found to be negative on 21 September (Table 1). Influenza H3 coinfection was discovered in her second respiratory specimen. Since she received basic biosafety training from her office, she was well aware of COVID-19 transmission including person-to-person infection and through respiratory droplets in the air. She also shared an interest in the COVID-19 vaccine, which she hopes would benefit both her family and community.

#### 3.1.3. Case 3

Case three was a hypertensive physician who reported a fever, headache and sore throat on 28 May (D1). Two days later, his PCR sample was found to be positive. He was isolated at home. Four days later, he became symptom-free. On 19 June, his follow-up sample was found negative. He was asymptomatic for the next 70 days. He reported fever, cold and low oxygen saturation (self-reported at 93%) on 12 August, and again was found to be PCR positive on 16 August. The case reported that within the past 14 days of his second episode, he had interaction with two COVID-19 relatives. During this second episode, he was tested for CBC, CRP, D-Dimer, LDH, a CT scan, and an echocardiogram. All were within normal limits except that a ground-glass appearance was evident on the chest CT and the CRP was elevated. After 12 days of illness, he became asymptomatic on 24 August (D90). On 29 August (D94) his PCR test was found to be positive. He was advised to isolate for another week without additional treatment and no further PCR testing was recommended. He reported having direct contact with COVID-19 positive patients and visiting them in the hospital is the riskiest way to acquire COVID-19 infection compared to exposure to an open environment like a wet market or workplace. He has a very positive attitude towards vaccinations.

#### 3.1.4. Case 4

Case four was an account officer with a history of recurrent asthma. He developed a fever on 1 June (D1) and tested positive for COVID-19 on 3 June. His fever subsided after three days. His next scheduled PCR test was negative on 25 June. Due to the nature of the job, case 4 reported that he frequently went to his office in the preceding 14 days using office transportation and also visited local markets for shopping. Eighty-five days after the relief of symptoms, he reported fever and cough on 29 August and was found PCR positive on 1 September. The staff clinic prescribed ivermectin, doxycycline, zinc for the second illness. His fever subsided 3 days after onset, but a dry cough persisted for seven days. On 21 September, his scheduled PCR test was negative. During his second episode, he was co-infected with influenza H3. His detailed treatment history and laboratory findings are depicted in Table 1 and Table 2. He had a basic understanding of how the virus spreads. For COVID-19 prevention, he emphasized handwashing with soap or sanitizer on a regular basis, maintaining social distance, and wearing a mask outside. He desired to get a COVID-19 vaccine as soon as possible to protect himself and safeguard his family.

#### 3.1.5. Case 5

Case five was a young doctor working at the Matlab hospital. She reported to the staff clinic on 9 May with a sore throat and cough starting on 7 May 2020. Both her father and son were previously diagnosed as COVID-19-positive and she was a close contact. On 12 May, her PCR test result was found positive. She was isolated at home and treated with azithromycin, hydroxychloroquine and zinc tablets. Fourteen days after initial positivity, consecutive tests, 24 h apart, were found to be negative. After a prolonged asymptomatic period (113 days), she was tested again, given she was unknowingly in close contact with a COVID-19-positive nurse at work in the preceding week. On 16th September, she tested positive for a second time. Two days after the second diagnosis, she reported a very severe headache, chest pain and a sore throat. She measured her oxygen saturation and it was low (90%). She was subsequently admitted to icddr,b hospital on 20 May. Her ECG, Chest X-ray, and D-dimer levels were tested and found to be within normal limits. As her symptoms improved over the day, she was discharged in the evening on the same date for home isolation. She also self-medicated with rivaroxaban tablets for the prevention of thrombotic events. She reported relief from symptoms on 23 September. Her follow-up PCR test conducted after 21 days was negative on 6th October. She reported that overconfidence, accompanied by carelessness, was the primary reason for getting the infection from a contaminated surface, or direct or indirect interaction with a COVID-19 positive individual. She reported that taking precautions such as using masks, gloves, hand sanitization, and maintaining a safe distance can reduce some risk of this infection. She was very enthusiastic about the COVID-19 vaccination because she thought it would minimize the risk of getting this disease.

## 4. Discussion

We reported a case series of five clinically and laboratory confirmed COVID-19 patients suffering a second episode of COVID-19 after a gap of over 70 days free of symptoms. The recurrent cases had largely mild symptoms in both the first and second episodes of illness (only one case had moderate symptoms during their second episode). Most of the samples had high CT values (>30) indicating a low viral load. Whole genome sequencing is essential for determining true reinfections; however, our attempt failed due to low viral loads in collected NP swabs. Therefore, it is still unclear whether these were due to true reinfections or possible persistent low-level of infection sustained by viable viral particles or extra viral RNA. Nevertheless, our small case series indicates that symptomatic SARS-CoV-2 recurrence can possibly occur among individuals who recovered from their initial illness.

Our institute data showed that approximately 1% of all staff to date had the second episode of COVID-19. The proportion of recurrent RNA positivity after recovering from COVID-19 in our study was lower than that reported by Camilla et al. (2020) in a meta-analysis, where the proportion was reported between 7 and 23% [17]. Several factors might explain the low percentage. Firstly, we followed a strict case definition for a patient to be included in our investigation with a combination of clinical symptoms, PCR positivity, at least a one-month gap between a negative test report and the reappearance of COVID-19 symptoms followed by the presence of viral RNA in a second PCR test. Secondly, we could not actively monitor and follow-up all first-time positive cases in person because of resource constraints, and the follow-up system depended on the passive reporting of symptoms by the staff to the clinic. As most employees worked from home, they were probably reluctant to report symptoms to the staff clinic in order to avoid social stigma [27]. Thus, we might have missed many mild to moderate symptomatic recurrences of COVID-19 among our study population. Thirdly, there is a high probability of asymptomatic reinfection [28] which was not captured in our surveillance system. Hence, the actual number of recurrences of SARS-CoV-2 RNA positivity in our population may have been underestimated. Consensus criteria for deciding true SARS-CoV-2 reinfection should be set as new findings accumulate. Finally, our cohort did not include a collection of blood samples to describe the immunological profile that could allow us to evaluate the immune status of the cases.

The protective role and longevity of antibody responses to the virus remain unanswered. Some recent reports suggest that neutralizing antibodies against the virus remain relatively stable for several months after infection. In line with another case series [29], it is not clear whether the virus can persist in some COVID-19 patients for a longer period and transmit to close contacts and later reappear as an apparent second illness with COVID-like symptoms. Therefore, we should still advise our staff to maintain social distance, wear masks and self-isolate as much as possible to prevent reinfection. Bangladesh has received 9 million doses of the COVID-19 vaccine, Covishield (AstraZeneca) from the Serum Institute of India and vaccinated a total of 3,682,152 individuals as of 7 March 2021 (Health Bulletin of DGHS, 7 March 2021). Given the global spread of new variants, it is yet to be explored whether mass vaccination against existing strains can reduce the incidence rate to sufficient levels required to halt the pandemic.

Two of our patients showed concomitant influenza virus infection during their second COVID-19 episode. Both SARS-CoV-2 and influenza virus have common signs and symptoms, so it is likely that many patients with respiratory tract symptoms can be co-infected with influenza (H3). The influenza positive cases were clinically indistinguishable from the other three cases. The role of influenza in altering the clinical course of COVID-19 is unclear, thus further studies will be required to explore the pros/cons of co-infection.

Most of our cases reported mild symptoms. The immunological memory against SARS-CoV-2 is not well established and it is uncertain whether the first infection can prevent re-infection [11]. Limited data suggest that SARS-CoV-2-specific CD4^+^/CD8^+^ T cells can persist for up to 3–5 months and IgG to the spike protein up to eight months in COVID-19 patients [13,14]. If true, all of our initial cases may have developed immunological memory which may have partially protected them during their second illness.

Though our study was based on systematically collected data and prospectively investigated by an experienced group of clinicians, epidemiologists, and virologists, it had several limitations. Firstly, we tried to compare whole genome sequence data obtained from the first and second episodes from individual cases. However, only two complete sequences (with 99% coverage) were recovered from the 10 isolates (Supplementary Figure S1). Therefore, we could not determine whether the virus detected was identical or a different variant. Secondly, we could not conclude whether these cases were true reinfections or just relapsed cases. It is possible that the virus in the first episode diminished in the upper respiratory tract, became PCR negative, but was sustained in other parts of the body such as the pharynx, trachea, lungs, heart, kidneys, intestines, brain, genitals, or in body fluids such as the cerebrospinal fluid, semen and breast milk [30]. A systematic review showed that the virus could be sustained in other samples, such as the stool, for a longer period of time after the nasopharyngeal swab was negative [31]. Natural infection should, in theory, offer a degree of protection against future reinfection. However, in reality, this is not always achieved, given the differences in innate immunity and host genetics. Influenza vaccines that include previous variants may not protect against a new variant, so reinfection is likely. A lack of acquired immunity may partially explain the observed COVID-19 recurrence, but further immunogenetic studies examining innate, acquired and concomitant immunity are required.

Active case finding and isolation, quarantine, regular hand washing, wearing face masks, cough etiquette, avoiding public meetings, and maintaining physical distancing are the steps of choice in the absence of necessary vaccination coverage. The efficacy of such initiatives, however, is largely dependent on public willingness to cooperate and their perceptions of risk towards COVID-19 [32]. All our COVID-19 positive cases were well aware of the coronavirus disease: how it spreads and what preventive measures should ideally be maintained to minimize the risk. Our findings support a previous study conducted in ten countries, including Asia, which found that people who had personal and direct experience with COVID-19 had significantly higher risk perceptions [33]. Although our whole genome sequencing was inconclusive, all five cases believed their second episode of the illness was reinfection because their sufferings were identical to (or even worse than) the first. Epidemiologically, they had the potential exposures before the second episode, and clinically their signs and symptoms were also compatible with COVID-19 infection.

## 5. Conclusions

Our findings suggest the need for the COVID-19 vaccination to halt community transmission as well as to continue all preventive measures such as mask, hand wash and social distancing. Additionally, an optimal post-recovery follow-up strategy to allow the safe return of COVID-19 patients to the workforce may be considered.

## Figures and Tables

**Table 1:** Clinical characteristics of the COVID-19 cases for both their first and second episodes of illness.

Patients Characteristics		First Episode				Clinically Symptom Free Days	Second Episode				
Case #	Past Medical History	Illness Onset Date (D1)	Clinical Feature	Treatment	First Clinical Recovery		Illness Onset Date	Clinical Feature	Treatment	Duration of Illness in Days	Outcome
1	HTN	D1 13 May	Fever, cough	Home isolation, A+D+I+Z+VD	D15	98	D112 2 September	Fever, cough, cold	Home isolation, D+I+Z+VD	18	Recovered
2	None	D1 19 May	Malaise	Home isolation, A+Z+HQ	D10	92	D 101 27 August	Sore throat, fever, cough, headache	Home isolation, D+Z+VD	21	Recovered
3	HTN	D1 28 May	F, H, S	Home isolation, D+I+Z+VD	D8	70	D77 12 August	F, C, Low oxygen saturation, pneumonic features in CT scan	Moxifloxacin, Amoxycillin with Clavulanic acid, P, F	12	Recovered
4	Asthma	D1 1 June	Fever	Home isolation, D+I+Z+VD	D6	85	D90 29 August	Fever, cold	Home isolation, D+I+Z	9	Recovered
5	HTN, HT	D1 7 May	Fever, cough	Home isolation, A+Z+HQ	D2	131	D135 18 September	S, H, Chest pain, Hospitalized	Home isolation, D+I+VD+R	6	Recovered

Note: for all patients, we considered the starting date of the illness (first day of symptom onset) as “Day One” (D1). Sign-symptoms: F = fever; H = headache; A=anosmia; J = joint pain; M = malaise; C = dry cough; S = sore throat; HTN = hypertension; DM = diabetes; HT = hypothyroidism; RhA = rheumatoid arthritis. Medications: A = azithromycin 500 mg tablet, once daily for 5 days; D = doxycycline 100 mg capsule, twice daily for 5 days; H = hydroxychloroquine 200 mg tablet, first day—two tab stat., from second day onwards—twice daily for 10 days; Z = zinc 20 mg tablet, twice daily for 14 days; VD = vitamin D3 2000 IU, one tablet on every alternate day for one month; M = montelukast 10 mg tablet, once daily for 10 days; R = rivaroxaban 10 mg tablet, once daily for 45 days; I = ivermectin 6 mg tablet, two tablets on day one only; amoxycillin with clavulanic acid (625 mg), eight hourly for 7 days; moxifloxacin, 400 mg tablet once daily for 7 days; P = tablet prednisolone for two weeks, starting at 16 mg daily for three days and then tapered dose; F = favipiravir 200 mg tablet, 1600 mg twice daily on day 1, 600 mg twice daily on day 2−10.

**Table 2:** Laboratory findings of the COVID-19 cases for both first and second episodes of illness.

Case #	First Episode			Gap between PCR Negative and PCR Positive Dates (days)	Second Episode				
	RT-PCR for SARS-CoV-2				RT-PCR for SARS-CoV-2			Other Investigations	
	Positive		Negative		Positive		Negative		
	Days from Symptom Onset	CT Value	PCR-Negative Day		Days from the first episode onset date	CT Value	PCR-Negative Day	Other Respiratory Viruses *	Other
1	D3 D15	38.3 33.7	D21	93	D114	34.8	D134	Negative	Chest X-ray-Normal D-Dimer-Normal
2	D5	21.7	D25	81	D106	34.8	D126	Influenza H3	ECG, BP normal
3	D3	34.5	D23	58	D81 D94	24.7 33.8	-	Negative	Ground glass appearance in CT scan chest, CRP level raised
4	D3	16.1	D25	68	D93	33.2	D113	Influenza H3	-
5	D6	36.8	D18, D20	115	D133	36.6	D153	Negative	ECG, Chest X-ray, D-Dimer all normal

* Influenza virus A and B, respiratory syncytial virus (RSV), parainfluenza (1, 2, 3), human metapneumovirus (hMPV), and adenovirus.

## Data Availability

Data cannot be shared publicly because they are confidential. Data are available from the respective department of icddr,b for researchers who meet the criteria for access to confidential data.

## Supplementary Materials

The following are available online at https://www.mdpi.com/article/10.3390/tropicalmed6020041/s1, Figure S1: Phylogenetic tree based on the SARS-CoV-2 genome, demonstrated homology between the icddr,b SARS-CoV-2 genomes (case numbers 2 and 4) and other reported SARS-CoV-2 genomes from Bangladesh retrieved from the GISAID database.

## References

[1] Cheng Z.J., Shan J. (2020). 2019 Novel Coronavirus: Where We Are and What We Know. Infection.

[2] Huang C., Wang Y., Li X., Ren L., Zhao J., Hu Y., Zhang L., Fan G., Xu J., Gu X. (2020). Clinical Features of Patients Infected with 2019 Novel Coronavirus in Wuhan, China. Lancet.

[3] Leung C. (2020). Clinical Features of Deaths in the Novel Coronavirus Epidemic in China. Rev. Med. Virol..

[4] Worldometer Coronavirus Update (Live): Cases and Deaths from COVID-19 Virus Pandemic. https://www.worldometers.info/coronavirus/.

[5] (2020). Bangladesh Preparedness and Response Plan for COVID-19. http://www.mohfw.gov.bd/index.php?option=com_docman&task=doc_download&gid=23359&lang=en.

[6] COVID-19. http://dashboard.dghs.gov.bd/webportal/pages/covid19.php.

[7] Transmission of SARS-CoV-2: Implications for Infection Prevention Precautions. https://www.who.int/news-room/commentaries/detail/transmission-of-sars-cov-2-implications-for-infection-prevention-precautions.

[8] Johansson M.A., Quandelacy T.M., Kada S., Prasad P.V., Steele M., Brooks J.T., Slayton R.B., Biggerstaff M., Butler J.C. (2021). SARS-CoV-2 Transmission From People Without COVID-19 Symptoms. JAMA Netw. Open.

[9] McAloon C., Collins Á., Hunt K., Barber A., Byrne A.W., Butler F., Casey M., Griffin J., Lane E., McEvoy D. (2020). Incubation Period of COVID-19: A Rapid Systematic Review and Meta-Analysis of Observational Research. BMJ Open.

[10] World Health Organization (2020). Laboratory Testing for Coronavirus Disease 2019 (COVID-19) in Suspected Human Cases.

[11] Grifoni A., Weiskopf D., Ramirez S.I., Mateus J., Dan J.M., Moderbacher C.R., Rawlings S.A., Sutherland A., Premkumar L., Jadi R.S. (2020). Targets of T Cell Responses to SARS-CoV-2 Coronavirus in Humans with COVID-19 Disease and Unexposed Individuals. Cell.

[12] Jeyanathan M., Afkhami S., Smaill F., Miller M.S., Lichty B.D., Xing Z. (2020). Immunological Considerations for COVID-19 Vaccine Strategies. Nat. Rev. Immunol..

[13] Iyer A.S., Jones F.K., Nodoushani A., Kelly M., Becker M., Slater D., Mills R., Teng E., Kamruzzaman M., Garcia-Beltran W.F. (2020). Persistence and Decay of Human Antibody Responses to the Receptor Binding Domain of SARS-CoV-2 Spike Protein in COVID-19 Patients. Sci. Immunol..

[14] Wajnberg A., Amanat F., Firpo A., Altman D.R., Bailey M.J., Mansour M., McMahon M., Meade P., Mendu D.R., Muellers K. (2020). Robust Neutralizing Antibodies to SARS-CoV-2 Infection Persist for Months. Science.

[15] Goldman J.D., Wang K., Röltgen K., Nielsen S.C.A., Roach J.C., Naccache S.N., Yang F., Wirz O.F., Yost K.E., Lee J.Y. (2020). Reinfection with SARS-CoV-2 and Failure of Humoral Immunity: A Case Report. medRxiv.

[16] The Prevalence of Long COVID Symptoms and COVID-19 Complications—Office for National Statistics. https://www.ons.gov.uk/news/statementsandletters/theprevalenceoflongcovidsymptomsandcovid19complications.

[17] Mattiuzzi C., Henry B.M., Sanchis-Gomar F., Lippi G. (2020). Sars-Cov-2 Recurrent Rna Positivity after Recovering from Coronavirus Disease 2019 (COVID-19): A Meta-Analysis. Acta Biomed..

[18] To K.K.-W., Hung I.F.-N., Ip J.D., Chu A.W.-H., Chan W.-M., Tam A.R., Fong C.H.-Y., Yuan S., Tsoi H.-W., Ng A.C.-K. (2020). COVID-19 Re-Infection by a Phylogenetically Distinct SARS-Coronavirus-2 Strain Confirmed by Whole Genome Sequencing. Clin. Infect. Dis..

[19] Tillett R.L., Sevinsky J.R., Hartley P.D., Kerwin H., Crawford N., Gorzalski A., Laverdure C., Verma S.C., Rossetto C.C., Jackson D. (2020). Genomic Evidence for Reinfection with SARS-CoV-2: A Case Study. Lancet Infect. Dis..

[20] Van Elslande J., Vermeersch P., Vandervoort K., Wawina-Bokalanga T., Vanmechelen B., Wollants E., Laenen L., André E., Van Ranst M., Lagrou K. (2020). Symptomatic SARS-CoV-2 Reinfection by a Phylogenetically Distinct Strain. Clin. Infect. Dis..

[21] Prado-Vivar B., Becerra-Wong M., Guadalupe J.J., Marquez S., Gutierrez B., Rojas-Silva P., Grunauer M., Trueba G., Barragan V., Cardenas P. (2020). COVID-19 Re-Infection by a Phylogenetically Distinct SARS-CoV-2 Variant, First Confirmed Event in South America. SSRN Electron. J..

[22] Criteria for Releasing COVID-19 Patients from Isolation. https://www.who.int/news-room/commentaries/detail/criteria-for-releasing-COVID-19-patients-from-isolation.

[23] Tomassini S., Kotecha D., Bird P.W., Folwell A., Biju S., Tang J.W. (2021). Setting the Criteria for SARS-CoV-2 Reinfection—Six Possible Cases. J. Infect..

[24] Marshall J.C., Murthy S., Diaz J., Adhikari N., Angus D.C., Arabi Y.M., Baillie K., Bauer M., Berry S., Blackwood B. (2020). A Minimal Common Outcome Measure Set for COVID-19 Clinical Research. Lancet Infect. Dis..

[25] Homaira N., Luby S.P., Hossain K., Islam K., Ahmed M., Rahman M., Rahman Z., Paul R.C., Bhuiyan M.U., Brooks W.A. (2016). Respiratory Viruses Associated Hospitalization among Children Aged <5 Years in Bangladesh: 2010–2014. PLoS ONE.

[26] Das P., Sazzad H.M.S., Aleem M.A., Rahman M.Z., Rahman M., Anthony S.J., Lipkin W.I., Gurley E.S., Luby S.P., Openshaw J.J. (2019). Hospital-Based Zoonotic Disease Surveillance in Bangladesh: Design, Field Data and Difficulties. Philos. Trans. R. Soc. B Biol. Sci..

[27] Mahmud A., Islam M.R. (2020). Social Stigma as a Barrier to Covid-19 Responses to Community Well-Being in Bangladesh. Int. J. Community Well-Being.

[28] Iwasaki A. (2020). What Reinfections Mean for COVID-19. Lancet Infect. Dis..

[29] Zheng K.I., Wang X.B., Jin X.H., Liu W.Y., Gao F., Chen Y.P., Zheng M.H. (2020). A Case Series of Recurrent Viral RNA Positivity in Recovered COVID-19 Chinese Patients. J. Gen. Intern. Med..

[30] Trypsteen W., Van Cleemput J., van Snippenberg W., Gerlo S., Vandekerckhove L. (2020). On the Whereabouts of SARS-CoV-2 in the Human Body: A Systematic Review. PLoS Pathog..

[31] Van Doorn A.S., Meijer B., Frampton C.M.A., Barclay M.L., de Boer N.K.H. (2020). Systematic Review with Meta-Analysis: SARS-CoV-2 Stool Testing and the Potential for Faecal-Oral Transmission. Aliment. Pharmacol. Ther..

[32] Asefa A., Qanche Q., Hailemariam S., Dhuguma T., Nigussie T. (2020). Risk Perception Towards COVID-19 and Its Associated Factors Among Waiters in Selected Towns of Southwest Ethiopia. Risk Manag. Healthc. Policy.

[33] Dryhurst S., Schneider C.R., Kerr J., Freeman A.L.J., Recchia G., van der Bles A.M., Spiegelhalter D., van der Linden S. (2020). Risk Perceptions of COVID-19 around the World. J. Risk Res..

