# Supplementary material for: A Case Series Describing the Recurrence of COVID-19 in Patients Who Recovered from Initial Illness in Bangladesh
Source: Trop Med Infect Dis. 2021 Mar 31;6(2):41. doi: 10.3390/tropicalmed6020041 (PMC8103235; doi:10.3390/tropicalmed6020041)
Supplement: Supplementary file 1 [file tropicalmed-06-00041-s001.pdf]

Tropical Medicine and Infectious Disease
MDPI

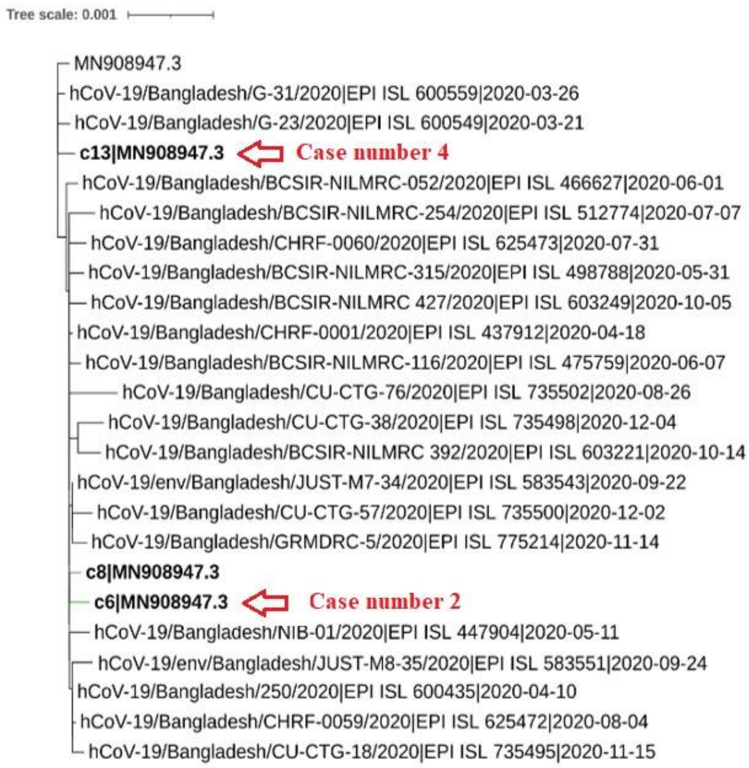

Figure S1. Phylogenetic tree based on the SARS-CoV-2 genome, demonstrated homology between the icddr,b SARS-CoV-2 genomes (case number 2 and 4) and other reported SARS-CoV-2 genomes from Bangladesh retrieved from the GISAID database.
